# Frailty and Different Exercise Interventions to Improve Gait Speed in Older Adults after Acute Coronary Syndrome

**DOI:** 10.3390/medicina57121344

**Published:** 2021-12-09

**Authors:** Aurelija Beigienė, Daiva Petruševičienė, Vitalija Barasaitė, Raimondas Kubilius, Jūratė Macijauskienė

**Affiliations:** 1 Department of Rehabilitation, Medical Academy, Lithuanian University of Health Sciences, Eivenių g. 2, LT-50161 Kaunas, Lithuania; daiva.petruseviciene@lsmuni.lt (D.P.); vitalija.barasaite@gmail.com (V.B.); raimondas.kubilius@lsmuni.lt (R.K.); 2 Department of Geriatrics, Medical Academy, Lithuanian University of Health Sciences, Eivenių g. 2, LT-50161 Kaunas, Lithuania; jurate.macijauskiene@lsmuni.lt

**Keywords:** frailty, cardiac rehabilitation, gait speed, resistance training, balance training, ischemic heart disease

## Abstract

*Background and Objectives*: The world’s population is rapidly aging, and it is estimated that, by 2050, every sixth person on earth will be older than 65 years. Around 30% of older adults entering cardiac rehabilitation (CR) meet the criteria of frailty. Frailty identification has not been included in the routine evaluation of CR patients yet, and there is a lack of evidence on what training regimen for improving physical performance in frail people is optimal. Therefore, the aim of our study was to determine the prevalence of frailty and to evaluate the effect of two different complementary training programs on the gait speed of older vulnerable and frail patients with acute coronary syndrome and mid-range-to-preserved left ventricular ejection fraction (≥40%) during short-term CR. *Materials and Methods*: This randomized controlled trial was conducted from January 2020 to September 2021. CR participants (*n* = 97) with a mean age of 73.1 ± 5.3 years were randomly allocated into three groups: control (CG, *n* = 32), intervention-1 (IG-1, *n* = 32) and intervention-2 (IG-2, *n* = 33). The patients of all three groups attended a usual inpatient CR program, and two intervention groups additionally received different resistance and balance training programs 3 days a week: the IG-1 underwent complementary training with traditional means of physical therapy, while the IG-2 underwent complementary training with mechanical devices. The mean CR duration was 18.9 ± 1.7 days. Frailty was assessed with the Edmonton Frail Scale, and the 5 m walk test was used to evaluate gait speed. *Results*: Frailty was determined in 37.1% of participants, and 42.3% met the criteria of being vulnerable. After CR, the gait speed of frail and vulnerable patients significantly improved in all three groups (*p* < 0.05). In the IG-2, slow gait speed was reversed to normal in the overwhelming majority of patients (*p* < 0.05), while the CG had the greatest proportion of patients who remained to be slow after CR (*p* < 0.05). *Conclusions*: A considerable part of patients entering CR are frail or vulnerable; therefore, it is of crucial importance to assess frailty status in all older people. All three CR programs improved gait speed in frail and vulnerable older patients with ischemic heart disease. Complementary resistance and balance training with mechanical devices more effectively reversed slow gait speed to normal during short-term CR.

## 1. Introduction

The prevalence of cardiovascular diseases increases with age, and this group of diseases is responsible for even 45% of all deaths in Europe [1]. Patients who are diagnosed with acute myocardial infarction and angina pectoris and who underwent coronary revascularization (percutaneous or surgical) are recommended to participate in cardiac rehabilitation (CR) by the American and European guidelines [2,3,4]. This multidisciplinary intervention is an individual program of aerobic and strength training, risk factor control, and lifestyle behavior change and education that is extremely beneficial for an aging society [5].

Advances in medicine are contributing to the growth of the older population worldwide, and projections indicate that, by 2050, every sixth person on earth will be older than 65 years (16%) compared with 1 out of 11 people (9%) in 2019 [6]. Due to a rapidly aging population, much attention is given to frailty. Frailty is a progressing, aging-related decline in physiological systems leading to loss of physical capacity reserves as well as maladaptive response to stressors and increased risk of various adverse health outcomes. It is caused by changes in cell and multiple organ system physiology, including sarcopenia, insufficient nutrition, and low level of physical activity [7]. Frailty is characterized by reduced muscle strength and endurance, decreased physiological functions, and consequently increased vulnerability to stress-related factors. Acute coronary syndrome or cardiac surgery leads to physiological stress. This condition is worsened by long-term bed rest and insufficient nutrition [8]. Therefore, almost 30% of older persons arrive to CR with reduced muscle strength and mass as well as limited physical performance [9,10], and this has a huge impact on balance, gait speed, and general ability to perform daily activities [11]. There is evidence that frailty is associated with adverse clinical outcomes such as falls, hospitalization, disability, need for nursing, and early mortality [12,13].

A recently conducted meta-analysis has shown that the overall estimated prevalence of frailty in Europe is 18%; however, in hospitals and nursing homes, the prevalence of frailty reaches 45% [14]. Frailty identification has not been included in the routine evaluation of patients with ischemic heart disease yet, and there is a lack of evidence that would allow for assigning any training of optimal type, intensity, and duration to such patients in order to improve their physical performance. Therefore, the aim of our study was to determine the prevalence of frailty and the impact of two different complementary resistance and balance training programs during short-term CR on gait speed of frail and vulnerable elderly patients who sustained acute coronary syndrome and had mid-range-to-preserved left ventricular (LV) ejection fraction (≥40%).

## 2. Materials and Methods

### 2.1. Study Design and Participants

The present study was carried out from January 2020 to September 2021 and is continuation of a larger clinical, randomized controlled trial conducted in a single rehabilitation center (Kulautuva Rehabilitation Hospital, Lithuanian University of Health Sciences, Kaunas, Lithuania) [15]. The study was approved by Kaunas Regional Biomedical Research Ethics Committee (permission No. BE-2-107, 19 December 2019) and was carried out following the guidelines of the World Medical Association Declaration of Helsinki. The trial was registered at ClinicalTrials.gov (No. NCT04768283).

All patients who are older and referred to CR after percutaneous coronary intervention or coronary artery bypass grafting (CABG) due to acute coronary syndrome were invited to take part in this study. The inclusion criteria were as follows: age ≥ 65 years; 6 min walk distance (6MWD) ≥ 150 m; LV ejection fraction ≥ 40%; and signed written informed consent. The exclusion criteria were as follows: combined CABG and valve surgery; severe cognitive impairment (Mini-Mental State Examination score of <18); implanted cardiac pacemaker; speech, vision, and hearing disorders; or other severe concomitant diseases that would not allow for active participation in the rehabilitation program and thorough examination.

### 2.2. Study Assessment

**Figure 1:**
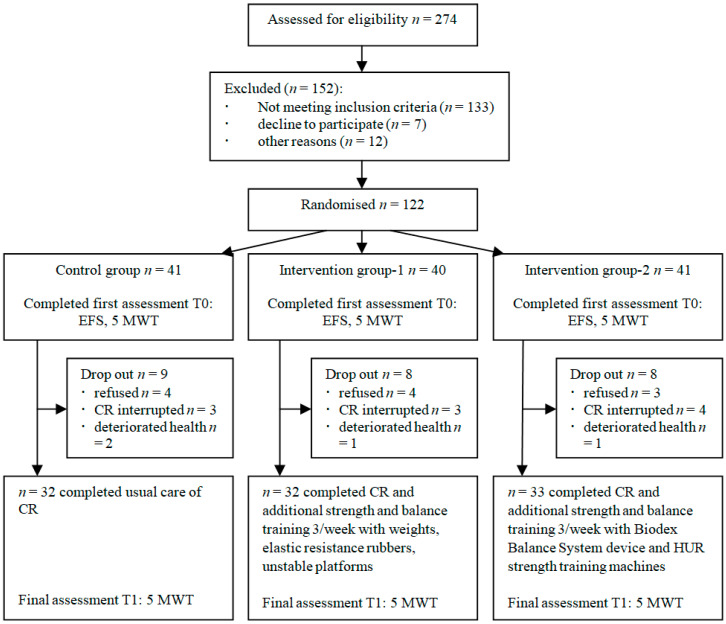
Study flowchart.

A total of 274 participants were recruited and assessed for eligibility to be enrolled in this study. Of these, 152 were excluded due to not meeting the inclusion criteria, declining to participate and other reasons, leaving with 122 participants who were randomly assigned to three groups: control (CG, *n* = 41), intervention-1 (IG-1, *n* = 40), and intervention-2 (IG-2, *n* = 41). After completion of the first assessment, 25 participants dropped out from the trial. The flowchart of the study is displayed in Figure 1.

Frailty was assessed with the Edmonton Frail Scale (EFS) [16,17,18]. The EFS is composed of 11 items, covering 9 components of frailty: cognition; general health status including the frequency of admissions to hospital; functional independence; social support; medication use (amount of medications and forgetfulness to take medications); nutrition; mood; urinary incontinence; and functional performance by performing a 3 m walking test, i.e., Timed Get Up and Go (TUG) test. Every item on the scale is rated by a score of 0 to 2; the maximal score achievable is 17, and this indicates severe frailty. During this study, the patients were classified based on their EFS score into the following categories as suggested on the official EFS website (Edmontonfrailscale.org): fit (score of 0 to 3), vulnerable (score of 4 to 5), and frail (score of 6 to 17) [15]. In addition, all of the participants passed a clinical evaluation of gait speed, using the 5 m walk test (5MWT). A gait speed of <1.0 m/s was defined as a slow gait speed and ≥1.0 m/s was defined as normal [19]. All of these assessments were conducted by a physical medicine and rehabilitation physician as well as a physical therapist before and after CR.

### 2.3. Study Interventions

The conventional CR program includes breathing exercises and aerobic training with an ergometer [20]. The participants allocated to the intervention groups along with conventional CR program received complementary balance and resistance training 3 times a week. The detailed complementary training programs and comprehensive study methodology are described in the previously published article [15]. Briefly, the IG-1 underwent complementary training with traditional means of physical therapy, while the IG-2 underwent complementary training with mechanical devices.

### 2.4. Statistical Analysis

Statistical analysis was carried out with Microsoft Excel 2013 and IBM SPSS Statistics 27.0 programs. Data were expressed based on their distribution: if data were non-normally distributed, they were expressed as median with interquartile range (IQR); if normally, as mean with standard deviation (SD). Categorical data were described by numbers and percentages. Three independent samples of normally distributed continuous data were compared using one-way analysis of variance (ANOVA), while the Kruskal–Wallis test was employed to compare three independent samples of non-normally distributed continuous data. Categorical data were compared with the chi-square test; the Fisher exact test was used when the frequency in at least one cell was small. For comparison of two dependent samples of non-normally distributed continuous data, the non-parametric Wilcoxon signed-rank test was used. The level of significance was set at *p* < 0.05.

## 3. Results

A total of 97 participants completed the study, and general characteristics of the study population are shown in Table 1. More than a quarter (26.8%) of the participants were women, and 73.2% were men. The mean age of participants was 73.1 ± 5.3 years. The groups were not homogeneous by age (*p* = 0.008), with the patients being significantly older in the CG compared with both intervention groups.

### 3.1. Frailty Prevalence

According to the EFS scores, 37.1% of participants were frail, 42.3% were vulnerable, and 20.6% were fit. All the groups were homogeneous regarding the median scores on the EFS. The results of frailty assessment with the EFS by the groups are depicted in Table 2.

### 3.2. Effectiveness of Cardiac Rehabilitation on Gait Speed

To evaluate the impact of CR on gait speed, only vulnerable (a score of 4 to 5 by the EFS) and frail participants (a score of ≥6 by the EFS) were selected (*n* = 77). Before CR, the groups were homogeneous by the mean gait speed (*p* = 0.941), and after CR, the mean gait speed of all vulnerable and frail participants increased significantly from 1.27 ± 0.32 to 1.43 ± 0.31 m/s (*p* < 0.001). Improvement in gait speed was documented in all three groups; however, a comparison of the change in gait speed among all three groups did not show any significant difference (Table 3).

Evaluation of gait speed in the groups revealed that, before CR, 77% of vulnerable and frail participants had normal gait speed (≥1 m/s), and the remaining 23% had a reduced gait speed (<1 m/s). In the CG, gait speed remained slow in 5 out of 10 participants after CR (*p* = 0.013), while in the IG-2, slow gait speed was reversed to normal in 6 out of 7 participants (*p* = 0.031).

## 4. Discussion

One of the aims of this study was to determine the prevalence of frailty among older patients (≥65 years) with acute coronary syndrome during CR. By using the multidimensional EFS to assess the prevalence of frailty, we found that 37% of patients were frail and that 42% met the criteria of being vulnerable. These results are attributable to the fact that, for the identification of frailty, not only the results of the TUG test, which showed 70% of participants completing the test faster than within 10 s, but also more aspects important to frailty, such as cognitive functioning, medication use, social support, mood, and general health status were evaluated [21,22]. It is worth noting that, currently, there is no universal tool for detecting frailty yet that could be considered the gold standard. Researchers utilize multiple frailty screening measures [23,24]; therefore, often, the assessments of frailty differ and are difficult to compare. Based on the findings of the systematic review in 2018, among four different study populations, the prevalence of frailty ranged from 11.8% to 44% [25]. In general, the first systematic review on the prevalence of frailty was published in 2012, and at that time, on average, 10.7% of community-dwelling older persons were frail and 41.6% were vulnerable [26]. In comparison, the most recent meta-analysis published in 2021 showed a greater prevalence of frailty and pre-frailty, being 16% and 45%, respectively [27]. The results of our study confirm that frailty is more common among inpatients [28,29] than in the general population [30,31].

One of the frailty-defining criteria is slowness, recognized by measuring gait speed [32]. Slow gait speed is associated with a higher risk of disability [33] and mortality [34]. Gait speed was found to be an independent predictor of adverse outcomes after cardiac, and every 0.1 m/s decrease in gait speed was related to an 11% relative increase in operative mortality [35]. Thus, an increase in gait speed would reduce the level of frailty.

The literature to date suggests that physical activity can effectively reverse frailty in older adults [36,37,38,39]. A combination of aerobic exercises with resistance training has been shown to be more effective at improving muscle strength, exercise performance, and cardiorespiratory fitness in older adults with ischemic heart disease compared with aerobic or resistance training applied separately [40]. Despite the evidence on the beneficial effects of multicomponent exercise training on muscle strength, gait speed, and balance of older frail and vulnerable patients [11,41,42], the type, duration, and intensity of exercise training for these patients have not been standardized. Therefore, another aim of our study was to evaluate the effect of two different complementary resistance and balance training programs on the gait speed of frail and vulnerable patients during CR. Our results show that gait speed of vulnerable and frail older patients with ischemic heart disease was improved both after the completion of the conventional CR program as well as complementary resistance and balance training 3 times per week. It is worth noting that complementary exercises with pneumatic technology-based HUR training devices and the Biodex Balance System were more effective at improving gait speed to normal (≥1 m/s) during short-term CR.

Unfortunately, we could not compare the results of our study with the findings of other studies worldwide as we failed to find other studies investigating frailty and patients with ischemic heart diseases who would be trained with the aim to improve gait speed. Only studies showing the benefit of long-term physical training for gait speed in frail older people have been published. The study by Kim et al. showed that nutrition supplementation in combination with 60 min strengthening and balance training given twice per week for 3 months improved gait speed in frail elderly women even by 14.7% [43]. Another study reported that 4-month resistance training had a positive impact on gait speed only in prefrail older adults, while frail older adults showed no improvement in gait speed [44]. Currently, there are no other studies proving the benefit of short-term physical training for gait speed in older people who are vulnerable and frail undergoing CR after acute coronary syndrome; therefore, we will continue our research in this field.

Some limitations of this study have to be acknowledged: (1) This study is a single-institution study; therefore, the results cannot be generalized to all patients who sustained acute coronary syndrome. (2) The inclusion criteria such as 6MWD ≥ 150 m and LV ejection fraction ≥ 40% could prevent the inclusion of many other frail patients. (3) This study included patients only with mid-range-to-preserved LV ejection fraction (≥40%); thus, the findings can be generalized only to these patients.

## 5. Conclusions

The findings of this study indicated that a considerable part of older patients (≥65 years) entering CR after acute coronary syndrome were found to be frail (37%) or vulnerable (42%). All three CR programs improved gait speed (5 MWT) in these patients; however, complementary resistance and balance training with mechanical devices 3 times per week had a greater effect on improving slow gait speed during short-term CR.

## Figures and Tables

**Table 1:** Sociodemographic and clinical characteristics of the study population.

Characteristic	All (*n* = 97)	IG-1 (*n* = 32)	IG-2 (*n* = 33)	CG (*n* = 32)	*p* Value *
Age, years	73.1 ± 5.3	72.6 ± 4.6	70.6 ± 4.3	74.6 ± 6.3	0.008
Sex, *n* (%)					
Female	26 (26.8)	8 (25)	11 (33.3)	7 (21.9)	0.558
Male	71 (73.2)	24 (75)	22 (66.7)	25 (78.1)	
Height, m	1.7 ± 0.1	1.7 ± 0.1	1.7 ± 0.1	1.7 ± 0.1	0.234
Weight, kg	80.1 ± 13.7	80.8 ± 12.3	83.8 ± 15.8	77.8 ± 12.3	0.220
Body mass index, kg/m^2^	27.9 ± 3.9	28.5 ± 3.4	28.4 ± 4.2	26.9 ± 4	0.176
Current smokers, *n* (%)	11 (11.3)	4 (12.5)	3 (9.1)	4 (12.5)	0.882
LV ejection fraction, %	47.7 ± 5.3	48.8 ± 5.1	46.8 ± 4.6	47.5 ± 56	0.311
CR duration, days	18.9 ± 1.7	19.6 ± 0.8	18.8 ± 1.6	18.3 ± 2.3	0.008
Comorbidities, *n* (%)					
Diabetes	15 (15.5)	7 (21.9)	2 (6.1)	6 (18.8)	0.174
Atrial fibrillation	18 (18.6)	6 (18.8)	7 (21.2)	5 (15.6)	0.845
Degenerative joint disease	6 (6.2)	1 (3.1)	4 (12.1)	1 (3.1)	0.219
Cancer	8 (8.2)	3 (9.4)	3 (9.1)	2 (6.3)	0.881
Treatment, *n* (%)					
Coronary artery by-pass graft	58 (59.8)	25 (78.1)	17 (51.5)	16 (50)	0.035
PTCA	39 (40.2)	7 (21.9)	16 (48.5)	16 (50)	
Medication, *n* (%)					
ACE inhibitor	78 (80.4)	22 (68.8)	27 (81.8)	29 (90.6)	0.085
Diuretic	70 (72.2)	26 (81.3)	24 (72.7)	20 (62.5)	0.246
Statin	93 (95.9)	30 (93.8)	32 (97)	31 (96.9)	0.761

Values are mean ± standard deviation unless indicated otherwise. * *p* value by the chi-square test (for categorical data) or ANOVA (for normally distributed continuous data). Bold *p*-values indicate statistically significant differences. IG-1, intervention group-1; IG-2, intervention group-2; CG, control group; LV, left ventricular; CR, cardiac rehabilitation; PTCA, percutaneous transluminal coronary angioplasty; ACE, angiotensin-converting enzyme.

**Table 2:** Results of the Edmonton Frail Scale.

Frailty Assessment	All (*n* = 97)	IG-1 (*n* = 32)	IG-2 (*n* = 33)	CG (*n* = 32)	*p* Value *
Total EFS score (0–17), median (IQR)	5 (4–6)	5 (4–6.8)	5 (3–7)	5 (4–6)	0.880
Frailty classification by EFS, *n* (%)					
Fit (0–3)	20 (20.6)	4 (12.5)	9 (27.3)	7 (21.9)	0.643
Vulnerable (4–5)	41 (42.3)	16 (50)	12 (36.4)	13 (40.6)	
Frailty (≥6)	36 (37.1)	12 (37.5)	12 (36.4)	12 (37.5)	
EFS items					
Cognition, *n* (%)					
Clock drawing test faultless	30 (30.9)	8 (25)	11 (33.3)	11 (34.4)	0.672
Clock drawing test with minor and major errors	67 (69.1)	24 (75)	22 (66.7)	21 (65.6)	0.210
One and more hospitalizations in the past year, *n* (%)	87 (89.7)	28 (87.5)	31 (93.9)	28 (87.5)	0.902
General health status, *n* (%)					
Excellent, very good, good	22 (22.7)	7 (21.9)	9 (27.3)	6 (18.8)	0.708
Fair, poor	75 (77.3)	25 (78.1)	24 (72.7)	26 (81.3)	0.470
Functional independence, *n* (%)					
Independent	76 (78.4)	27 (84.4)	27 (81.8)	22 (68.8)	0.265
Require help in two and more activities	21 (21.6)	5 (15.6)	6 (18.2)	10 (31.3)	0.944
Social support, *n* (%)					
Always	91 (93.6)	31 (96.9)	31 (93.9)	29 (90.6)	0.957
Sometimes, never	6 (6.2)	1 (3.1)	2 (6.1)	3 (9.4)	NA
Medication use, *n* (%)					
Amount ≥ 5	80 (82.5)	27 (84.4)	28 (84.8)	25 (78.1)	0.916
Forgetfulness	14 (14.4)	5 (15.6)	3 (9.1)	6 (18.8)	NA
Nutrition–weight loss, *n* (%)	22 (22.7)	8 (25)	8 (24.2)	6 (18.8)	0.808
Mood–sad, depressed, *n* (%)	30 (30.9)	11 (34.4)	10 (30.3)	9 (28.1)	0.860
Urinary incontinence, *n* (%)	7(7.2)	3 (9.4)	2 (6.1)	2 (6.3)	NA
Functional performance by the TUG test, *n* (%)					
0–10 s	68 (70.1)	23 (71.9)	23 (69.7)	22 (68.8)	0.962
11–20 s	29 (29.9)	9 (28.1)	10 (30.3)	10 (31.3)	0.607

* *p* value by the chi-square test (for categorical data). IG-1, intervention group-1; IG-2, intervention group-2; CG, control group; EFS, Edmonton frail scale; TUG, Timed Get Up and Go test; IQR, interquartile range; NA, not applicable.

**Table 3:** Gait speed before and after cardiac rehabilitation.

Parameter	IG-1 (*n* = 28)		IG-2 (*n* = 24)		CG (*n* = 25)		*p* *	
	T0	T1	T0	T1	T0	T1	Within groups	Among groups
Gait speed, m/s	1.23 (1.02–1.45)	1.41 (1.18–1.61)	1.37 (0.92–1.59)	1.56 (1.3–1.66)	1.35 (0.94–1.56)	1.53 (1–1.68)	<0.05	0.100

Values are medians (interquartile range). * *p*-value by Kruskal–Wallis test. IG-1, intervention group-1; IG-2, intervention group-2; CG, control group.

## Data Availability

The data presented in this study are available from the corresponding author upon request. The data are not publicly available due to ethical restrictions and data protection policies.
